# Atypical Leydig Cell Tumor in a Young Child

**Published:** 2013-06-26

**Authors:** Ciro Esposito, Maria Escolino, Alessandro Settimi

**Affiliations:** Department of Pediatrics (Section Surgery), Federico II University of Naples, Via Pansini 5 80131, Naples Italy; Department of Pediatrics (Section Surgery), Federico II University of Naples, Via Pansini 5 80131, Naples Italy

**Dear Sir,**

 The Leydigioma is a rare tumor of the testicle. It develops from the proliferation of the interstitial cells or Leydig cells, and compete with the endocrine activity of this organ.[1] It usually arises between 20 year and 50 year of life, and rarely reported in children.[2] International literature is rich about the management of testicular tumors in adults and often recommends radical orchidectomy. Few reports of pediatric Leydig cells tumors exist in literature.[2, 3] The tumor cells produce androgen hormones and manifests with precocious physical and sexual development.[4] Rarely the tumor is asymptomatic. We report a case of asymptomatic Leydig cell tumor managed by testis sparing surgery.

A 5-year-old boy was admitted for the management of left inguinal hernia. Clinical examination of right testicle showed 3 ml volume and normal location with an incidental palpation of a right testicular mass; the left testicle was of 2 ml volume with normal position. Testicular ultrasound of right testicle showed a 5 mm hyperechoic nodule at lower pole. The hormonal assessment (including LH, FSH, testosterone, 17-hydroxyprogesteron and dehydroepiandrostenedione) and the tumor markers (a-FP, Beta HCG, CA-125 and CEA) were unremarkable. Magnetic resonance imaging of the lower abdomen and the pelvic cavity was normal and moreover it showed a 5 mm area of hypointensity located at the lower pole of right testis. The tumor was approached via trans-scrotal approach under caudal anesthesia. There was a gray brown colored nodule of 5 mm inside the testicular parenchyma. (Fig.1, 2) The nodule was enucleated with gentle electro-cautery. We did not conduct a peripheral frozen biopsy due to the well circumscribed aspect of the lesion. In the same session left inguinal herniotomy was done. The postoperative period was uneventful. The histological report demonstrated the diagnosis of a benign well differentiated Leydig cell tumor. The patient is being followed-up with clinical examinations, ultrasonography, chest x-rays, and tumor markers twice a year and doing fine.

**Figure:**
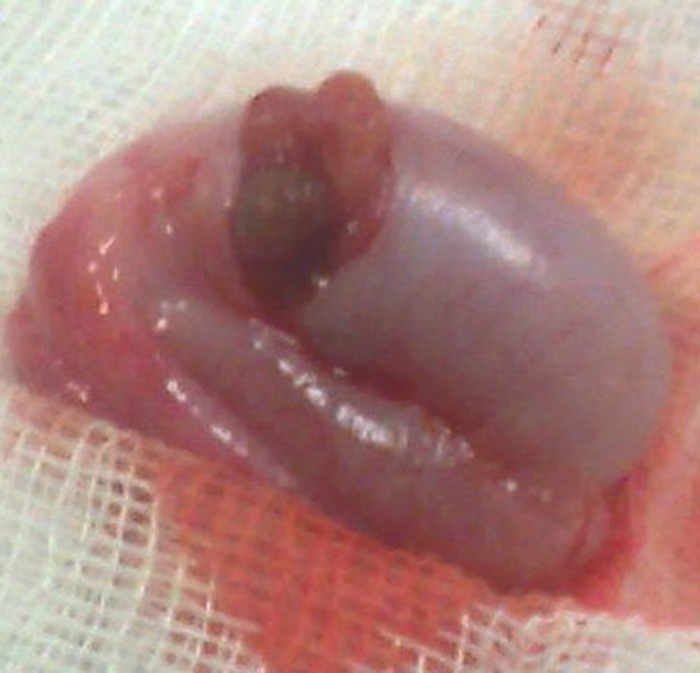
Figure 1: Operative picture shows a brown colored mass located at the inferior testicular pole.

**Figure:**
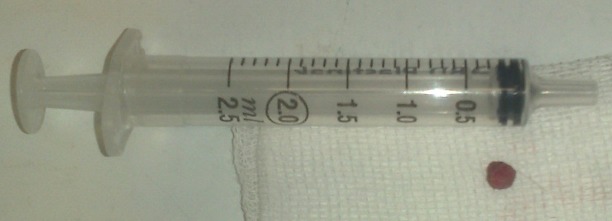
Figure 2: The tumor of 5mm in diameter was completely resected.

Leydig cell tumors account for 1.5% of all testicular tumors and 39% of gonadal stromal tumors.[1, 3] The diagnosis is made by raised plasma androgens and estrogens, follicle-stimulating hormone (FSH), luteinizing hormone (LH), and tumor markers together with scrotal ultrasound although the definitive diagnosis is histological.[2] Our case was unusual as related hormone studies were normal. Testicular computed tomography (CT) scan is not recommended due to the high radiation dose and can be considered superfluous for the high specificity and sensitivity of the ultrasound examination; eventually an MRI can be performed as we did in our case to exclude metastasis.[1, 6] In adults the symptoms are usually caused by the concurrent production of estrogens and corticosteroids e.g., appearance of gynecomastia that constitutes the first sign of this tumor before the testicular volume increases.[2, 3] In our case the patient was asymptomatic and the lesion was discovered incidentally. Therefore a careful palpation of testis, as happened in our patient, was extremely important and useful for the early diagnosis of this tumor.

Treatment is surgical. Previously orchidectomy was the option. In the recent literature there is strong recommendation of tumor enucleation as the most appropriate treatment for testicular benign lesions.[3, 5] We have opted for testis sparing surgery and our patient showed a recurrence free follow up of more than three years (to this date).

In conclusion, Leydig cell tumor may not always present with hormonal manifestations. Testicular sparing surgery by simple enucleation is a safe option for these benign lesions.

## Footnotes

**Source of Support:** Nil

**Conflict of Interest:** None declared

